# Overactivation of the IGF signalling pathway in osteosarcoma: a potential therapeutic target?

**DOI:** 10.1002/cjp2.191

**Published:** 2020-12-08

**Authors:** Baptiste Ameline, Michal Kovac, Michaela Nathrath, Maxim Barenboim, Olaf Witt, Andreas H Krieg, Daniel Baumhoer

**Affiliations:** ^1^ Bone Tumour Reference Centre at the Institute of Pathology University Hospital Basel, University of Basel Basel Switzerland; ^2^ Faculty of Informatics and Information Technologies Slovak University of Technology Bratislava Slovakia; ^3^ Department of Pediatrics and Children's Cancer Research Center, Klinikum rechts der Isar Technical University of Munich, School of Medicine Munich Germany; ^4^ Pediatric Hematology and Oncology Klinikum Kassel Kassel Germany; ^5^ Coordinator INFORM Program, Hopp Children's Cancer Center, German Cancer Research Center University Hospital Heidelberg Heidelberg Germany; ^6^ Bone and Soft tissue Sarcoma Center University of Basel, University Childrens Hospital (UKBB) Basel Switzerland

**Keywords:** osteosarcoma, IGF1R, targeted treatment, chromoanagenesis

## Abstract

Osteosarcoma is the most common primary malignant bone tumour in children and adolescents. More than a third of patients do not respond to standard therapy and urgently require alternative treatment options. Due to a high degree of inter‐ and intra‐tumoural genomic heterogeneity and complexity, recurrent molecular alterations that could serve as prognostic predictors or therapeutic targets are still lacking in osteosarcoma. Copy number (CN) gains involving the *IGF1R* gene, however, have been suggested as a potential surrogate marker for treating a subset of patients with IGF1R inhibitors. In this study, we screened a large set of osteosarcomas and found specific CN gains of the *IGF1R* gene in 18 of 253 (7.1%) cases with corresponding IGF1R overexpression. Despite the discouraging results observed in clinical trials in other tumours so far, focusing only on selected patients with osteosarcoma that show evidence of IGF pathway activation might represent a promising new and innovative treatment approach.

## Introduction

The insulin‐like growth factor (IGF) signalling pathway has crucial physiological implications for the homeostasis of bone [1]. IGF‐1 plays a key role in longitudinal bone growth through its interaction with its receptor in response to growth hormone exposure. IGF‐1 receptor (IGF1R) activation induces the recruitment and stimulation of signalling adaptor proteins such IRS‐1/2 and SHC that trigger the PI3K/AKT and the RAS/MAP kinase signalling cascades [2].

Deregulation of IGF1R expression on the other hand can contribute to cancer progression and has been described in osteosarcoma previously [3]. In this context, the PI3K/AKT pathway seems to be over‐activated during early tumour development and pulmonary spread whereas RAS/MAPK pathway activation could rather play a role at later stages of pulmonary dissemination [4]. Interestingly, overexpression of IGF1R also has been shown in canine osteosarcoma and to strongly correlate with tumour stage and adverse outcome [5]. As a consequence, numerous academic research groups and companies developed neutralising antibodies (anti‐IGF1R), small molecule tyrosine kinase inhibitors (TKIs) or small interfering RNAs (si‐RNAs) to target IGF1R as a molecular treatment strategy [6, 7, 8, 9].

Unfortunately, IGF1R inhibition using monoclonal antibodies (e.g. cixutumumab, dalotuzumab and robatumumab) or tyrosine kinase inhibitors (linsitinib) has not succeeded in inducing durable remissions in bone sarcomas or other tumour types so far [10]. Escape mechanisms involving the heterodimerisation of IGF1R with either the human epidermal growth factor receptor (EGFR) or the insulin receptor (IR) have been described as underlying causes [11, 12]. Alternative strategies are currently under investigation to prevent IGF1R activation, such as targeting its ligands (IGF1 and IGF2) with neutralising antibodies or combining anti‐IGF1R treatment with additional inhibitors of the PI3K/AKT and/or the RAS/MAP kinase cascades to avoid adaptive responses [13, 14]. It seems crucial to identify biomarkers that can help to discriminate potential responders from non‐responders to IGF1R inhibition. An increased sensitivity to this approach has been observed in cases of fusion gene‐driven tumours such as *MYB‐NFIB* related adenoid cystic carcinomas of the salivary glands or Ewing sarcoma with gene fusions involving members of the FET family of genes [15]. The authors show that, in both cases, the pathogenicity of the fusion protein directly affects the activation of the IGF1 receptor.

In the largest sequencing study of osteosarcoma so far, 8 of 112 patients with osteosarcoma (7%) presented with high copy gains of *IGF1R* using whole exome sequencing data. When using FISH in a smaller subset of cases, the percentage of amplified cases was even higher (12/87, 14%) [16]. Further analyses revealed the presence of indels among additional members of the IGF gene family (*IGFBP5*, *IGF2R*) in three other patients. The authors therefore concluded that the IGF signalling pathway might be therapeutically exploitable in a subgroup of patients with osteosarcoma.

The purpose of this study was to validate these results in a larger set of 253 osteosarcomas and therefore add evidence for a potentially new targeted treatment option.

## Methods

### Sample collection

Tumour samples from 253 patients with osteosarcoma were included. All samples were re‐evaluated by an experienced bone pathologist who confirmed the diagnosis and a tumour content >50% per sample. The male to female ratio was 51:49, the age range was 2–59 years (median: 16.5 years). Numerous samples evaluated (*n* = 41) in this study were provided by the INFORM program [17]. All tumours represented conventional high‐grade osteosarcomas. Ethical approval was given by the Ethikkommission beider Basel (reference 274/12).

### FISH study

FISH was performed using a Kreatech IGF1R probe (Ref KBI‐40116, red; Leica Biosystems, Germany) and an Abbott centromere 15 control probe (Vysis CEP15 15p11.2, Ref 06J54‐015, light blue; Abbot, Switzerland) according to the manufacturer's instructions.

### DNA sequencing

For whole genome sequencing (WGS) (*n* = 109), paired‐end libraries from fresh frozen tumour samples and paired‐blood DNA were prepared using the Agilent SureSelectXT HumanV5 kit for WGS. These were sequenced together with a tumour complementary DNA on an Illumina HiSeq2500 (paired‐end 100 bp).

For whole exome sequencing (WES) (*n* = 96), exome capture was performed using the Agilent SureSelect kits (version 4) from fresh frozen material. Samples were sequenced using the Illumina HiSeq 2000 platform as paired 100‐bp reads with Chemistry version 3.0. Sequencing reads were mapped to the GRCh37 human reference genome using HISAT2. A more detailed protocol can be found in either Worst *et al*. [17] (WGS) or Kovac *et al*. [18] (WES). Information on how to access publicly available sequencing data included in this study is given in supplementary material, Table S1.

### Variant calling

Raw sequencing reads were quality‐checked (fastqc ver. 0.11.7), duplicate‐removed (Picard tools ver. 2.9) and mapped onto the hs37d5 version of the human genome. The GATK pipeline (ver. 3.7) was used to perform base‐quality score recalibration and variant calling. We used the 2019 versions of VEP databases to annotate variants (ver. 95). Germline or somatic origins of the variants and indels were determined based on their presence or absence in the matched tumour‐free tissue. We applied the following exclusion filters to somatic variants: (1) variant present in any read from paired normal sample; (2) fewer than 10 reads in total at the variant site in the normal sample; (3) fewer than eight reads in total in the tumour; (4) variant present in fewer than three reads in the tumour and variant allele frequency below 20%; (5) presence of the variant in the Exome Aggregation Consortium dataset at a frequency >2% and (6) variant annotated as ‘common variant’ in the ‘FILTER’ category of VEP.

### Structural variants

Copy number (CN) aberrations were detected by segmenting log2 values extracted from either WGS (*n* = 109) or WES (*n* = 19) analysis using Nexus 10.0 software (BioDiscovery) or the R package ‘cnvkit’. The thresholds used to call an amplification or a deletion were log_2_(ratio) ≥ 1 and log_2_(ratio) ≤ −1, respectively. CN recurrence analyses were performed using the GISTIC2.0 module from the Broad Institute with the following parameters: broad = 1, ta = 0.6, td = 0.6, qvt = 0.05, cap = 3, maxseg = 2500, conf = 0.95. All other parameters were used with their default values. To identify structural rearrangements, the sequence data were analysed using the structural variant callers TIDDIT and BreakDancer.

### RNA sequencing

Sequencing libraries were prepared using the TruSeq RNA Sample Preparation Kit v2 (Illumina) following the manufacturer's instructions. Briefly, mRNA was purified from 1 μg of total RNA using oligo(dT) beads. Then, poly(A)+ RNA was fragmented to 150 bp and converted to cDNA. The cDNA fragments were then end‐repaired, adenylated on the 3′ end, adapter ligated and amplified with 12 cycles of PCR. The final libraries were quantified using Qubit (Invitrogen) and a size profile analysis was done using an Agilent 2200 Tapestation (Agilent Technologies). The libraries were subjected to two lanes of 2x100 bp paired‐end sequencing on the Illumina HiSeq2500 in rapid run mode according to the manufacturer's protocol using the TruSeq SBS Kit v3 (P/N: FC‐4013001). A more detailed protocol can be found in Worst *et al*. [17].

### Differential expression analysis

The alignment, quantification, normalisation, and differential expression analysis were performed by HISAT2 v2.1.0, StringTie v1.3.3 and DESeq2 v1.2 using the GRCh37 reference genome and the corresponding genomic annotation file. False discovery rate (also called adjusted *P* value) < 0.05 was set as a threshold to identify differentially expressed genes. Gene Set Enrichment Analysis was evaluated against the hallmark gene sets, available on the Molecular Signatures Database (MSigDB), using the fgsea R‐package [19].

### Fusion transcript detection

ChimeraScan and deFuse algorithms were used to detect chimeric transcript from RNA‐seq fastq files (*n* = 114). Predicted fusions were filtered out based on the presence of chimeric spanning or encompassing reads. The sequences of reads spanning a gene of interest were then blasted against the human transcriptome in order to exclude any ambiguity concerning the involved partners.

## Results

Our study aimed to identify mutations affecting the IGF family of genes (*IGF1*, *IGF1R*, *IGF2*, *IGF2R*, *IGFBP1*, *IGFBP2*, *IGFBP3*, *IGFBP4*, *IGFBP5*, *IGFBP6*) and their functional impact at the transcriptomic level. We searched for point mutations in the coding sequence (substitutions and indels), somatic copy number variations (SCNVs) and structural variants (breakpoints, gene fusions, and inversions) and included also gene set enrichment analyses.

We first searched for point mutations and indels, considering only non‐synonymous single nucleotide variants (SNVs) and indels in the coding sequence of IGF family genes. In 96 tumours investigated by high‐coverage exome sequencing, we detected missense mutations in: *IGF1R* (*n* = 1), *IGF2R* (*n* = 1) and *IGFBP5* (*n* = 2). To date, none of those have yet been associated with any functional impact (ClinVar). Assessment of pathogenicity using *in silico* methods (SIFT, PolyPhen, VEP) suggested that *IGF2R* and *IGFBP5* alterations were probably benign whereas the *IGF1R* missense mutation seems deleterious (see supplementary material, Table S1).

Somatic copy number variations (SCNVs) of the IGF gene family affected 9 of 134 tumours (6.7%, Table 1) using DNA sequencing data. Focusing only on the *IGF1R* gene, CN gains were detected in seven patients (5.2%). Twelve tumours showed CN gains (0.6 < log_2_(ratio) < 0.9) below the threshold used for defining amplification (log_2_(ratio) ≥ 1) and might indicate a subgroup of tumours harbouring sub‐clonal amplifications of *IGF1R*. The two remaining variations were CN losses of *IGF2* and *IGFBP6*. Interestingly, the amplitude of the SCNVs correlated inversely with its size (Table 1). Missense mutations and CN alterations were mutually exclusive and occurred preferentially in samples from metastases whereas our dataset mainly contains primary tumour samples (*P* value = 0.02).

**Table 1:** Characteristics of copy number variations observed among the IGF family genes.

Sample ID	P34	P14	P17	ST03	OS103	P02	P37	ST10	P258
Gene	*IGF1R*	*IGF1R*	*IGF1R*	*IGF1R*	*IGF1R*	*IGF1R*	*IGF1R*	*IGF2*	*IGFBP6*
Log_2_(ratio)	4.4	3.4	2.6	1.5	1.2	1.0	1.0	−1.2	−2
CN gain/loss*	+40.2	+19.2	+10.2	+3.6	+2.6	+2.0	+2.0	−1.1	−1.5
Size (Mbp)	1.5	2.2	2.3	4.3	6.0	8.5	8.6	10.8	0.2
Sample type	MET	MET	MET	MET	PRI	MET	MET	MET	PRI

All CN variations with an absolute log_2_(ratio) ≥ 1 are listed. Copy number gains (‘+’) or losses (‘−’) are estimated in absolute number (*) in comparison to a diploid cell. Mbp, million base pair; MET, metastasis; PRI, primary tumour.

Among the tumours with *IGF1R* CN gains, several presented with highly recombined genomes involving hundreds of inter‐ and intra‐chromosomal rearrangements. Some cases showed chromothripsis patterns which are well known in osteosarcoma. However, none of the structural rearrangements involved the *IGF1R* gene or any other of the IGF genes studied. In line with these observations, no gene fusions involving members of the IGF gene family or any other known chimeras [20] resulting in *IGF1R* activation were detected (*n* = 114).

**Figure 1:**
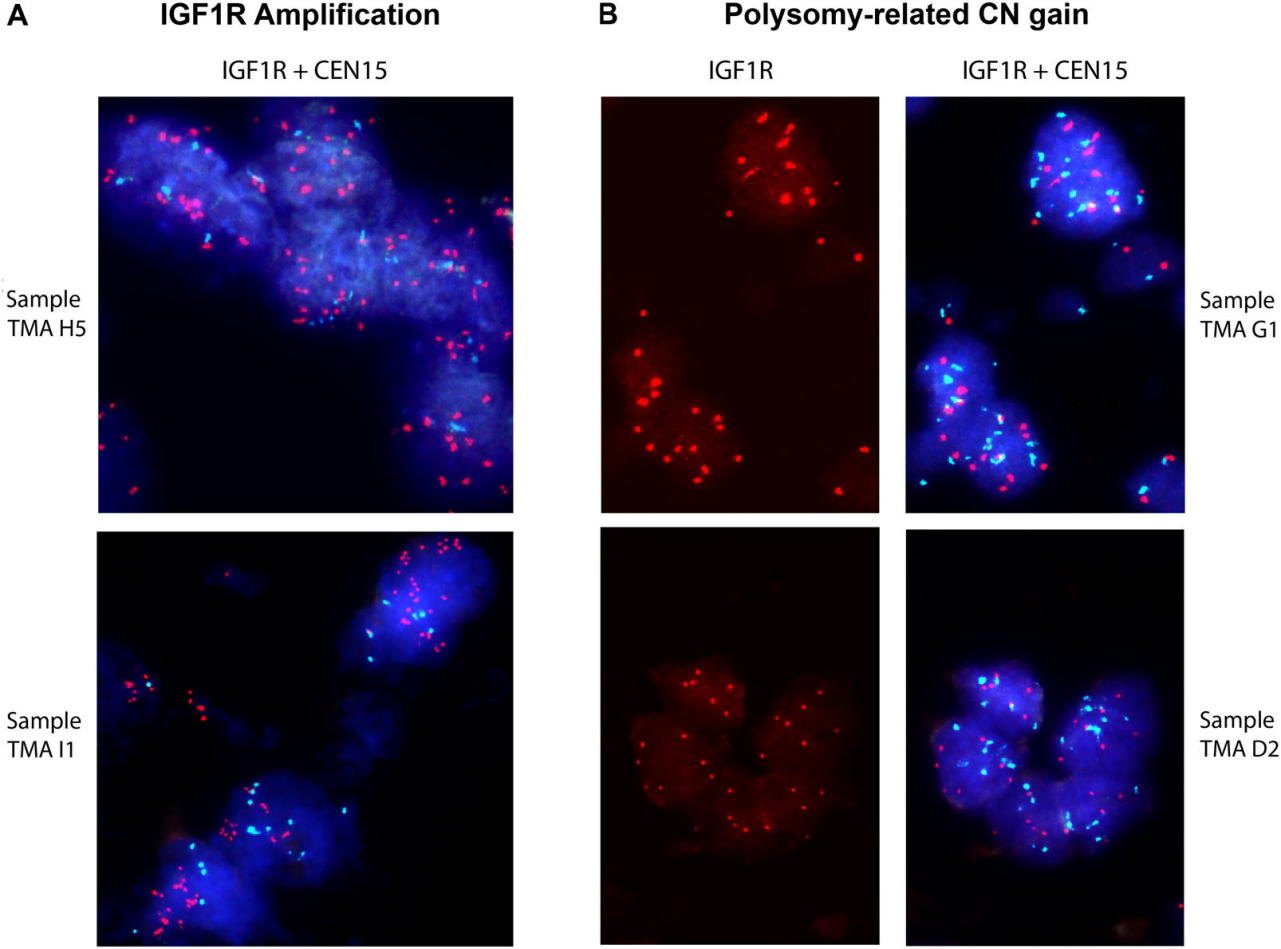
FISH analysis. (A) Osteosarcoma showing amplification of *IGF1R* (ratio of gene‐specific and centromeric probes >2). (B) Tumours demonstrating non‐specific polysomy of chromosome 15 (both signals increased but ratio <2). The *IGF1R* gene probe is coloured red and the centromeric control probe for chromosome 15 light blue.

In order to corroborate these results obtained by DNA sequencing, an independent set of 119 FFPE tumour samples was examined for *IGF1R* CN alterations using FISH (fluorescent *in situ* hybridisation). A total of 11 tumour samples revealed amplifications of *IGF1R* defined by a ratio between the *IGF1R* and the centromeric probe >2 (9.2%, Figure 1A). The highest ratio observed was 6. In numerous samples we observed a polysomy of chromosome 15 (multiple signals of both the gene‐specific and centromeric signals, emphasising the importance of normalising the IGF1R signal by using a centromeric control probe, Figure 1B).

**Figure 2:**
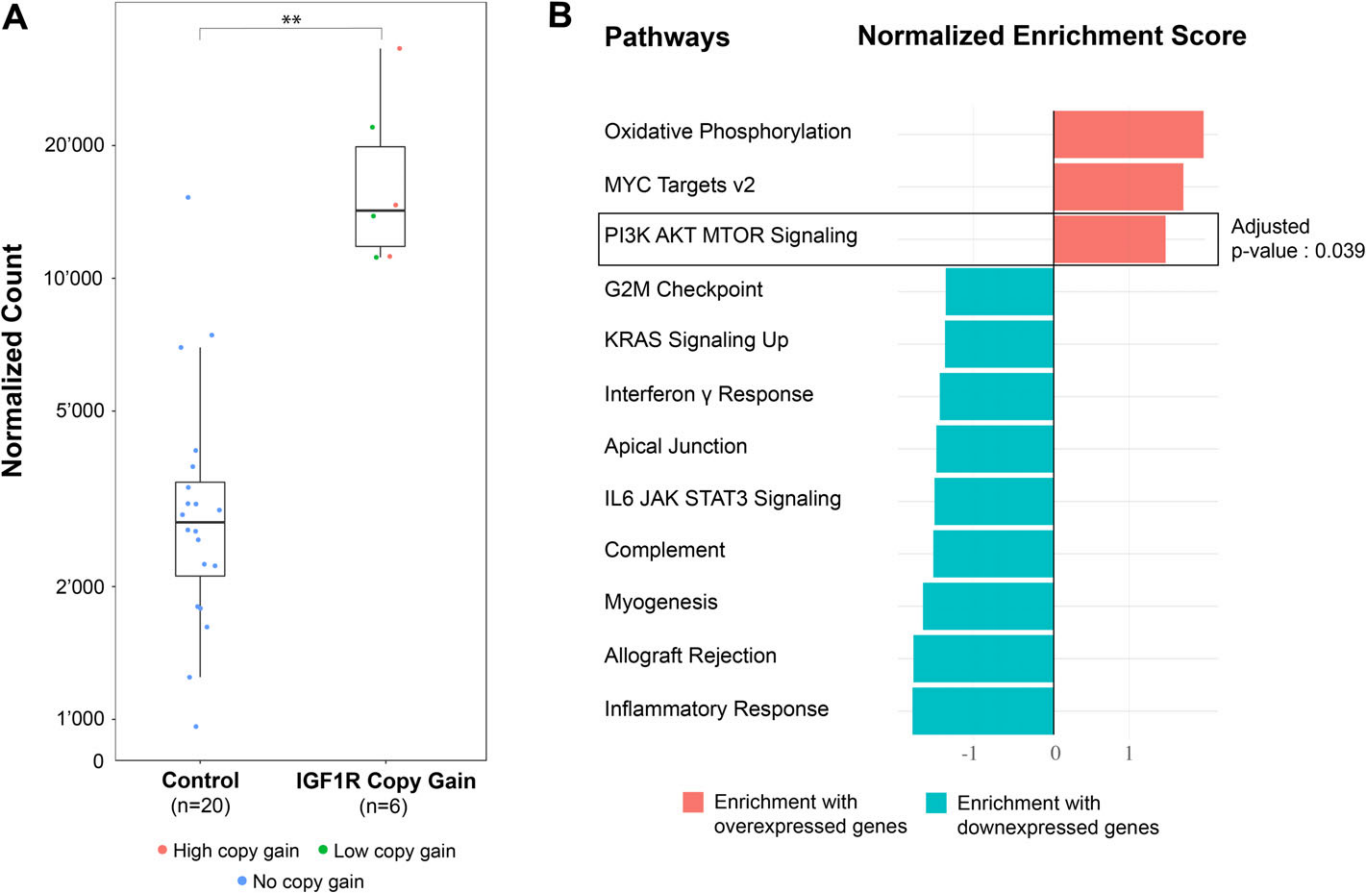
Impact of *IGF1R* amplification on transcription. (A) Comparing the *IGF1R* expression in tumours with (*n* = 6) and without (*n* = 20) *IGF1R* amplification revealed a statistically significant association using a Wald's test (log_2_ fold change: 2.17; *P* value: 10^−7^; adjusted *P* value: 10^−4^). Samples with a high *IGF1R* CN gain (ratio > 4) and a low *IGF1R* CN gain (ratio > 2) are respectively coloured in red and green. (B) Gene set enrichment analysis using the curated database ‘Hallmark’. Among the most differently expressed genes between the two groups, there was a significant over‐representation of genes involved in the PI3K‐AKT signalling pathway (*P* value: 0.009; adjusted *P* value: 0.039).

In a next step, we continued to evaluate the functional impact of SCNVs within the IGF signalling pathway. RNA sequencing was conducted in six of seven samples showing an IGF1R CN gain in DNA sequencing data. A set of 20 tumours without alterations in the IGF gene family served as a control group. Differential expression analysis was performed between both groups and IGF1R ranked among the 10 most differentially expressed genes (*P* value: 10^−7^, adjusted *P* value: 10^−4^) with a log_2_ fold change of 2.2 (Figure 2A). Notably, the overexpression of IGF1R was similar for all cases with increased CN and unaffected by the actual number of *IGF1R* copies gained. We then correlated the *IGF1R* CN with the signalling pathways of the ‘Hallmark’ database, using a gene set enrichment analysis (GSEA). Several gene sets were found to be significantly enriched among the most differentially expressed genes (Figure 2B), such as the ‘MYC targets v2’ and the ‘PI3K/AKT/mTOR’ signalling pathways (*P* value: 0.009, adjusted *P* value: 0.039).

Activation of the PI3K/AKT/mTOR axis was to be expected and MYC is a well known driver of osteosarcoma [21]. Therefore, both of these gene sets were further investigated with the aim of detecting recurrent mutations (SNVs and SCNVs) among these potential downstream effectors of the IGF1R axis (see supplementary material, Figures S1 and S2). In order to narrow down the investigation to functionally relevant alterations, we performed a CN recurrence analysis using the GISTIC2.0 module (see supplementary material, Figure S3). Among the recurrently amplified/deleted loci, we selected only those containing genes of either the two mentioned pathways or driver genes of the COSMIC Cancer Gene Census (see supplementary material, Table S1). Then, we selected the remaining SCNVs co‐occurring with the IGF alterations. Four candidate loci passed all filters: 6p25.3 (*IRF4*), 12p12.3 (*CDKN1B*/*KRAS*), 17p11.2 (*FLCN*/*MAP2K3*) and 17p13.1 (*GAS7*). Finally, we investigated the impact of *IGF1R* amplification on the transcription of each of these genes (see supplementary material, Table S1). No significant associations were found with either the amplification of *MYC* or its transcription. Among the three differentially expressed genes (*CDKN1B*, *FLCN* and *MAP2K3*), two are directly regulated by AKT (*CDKN1B* and *MAP2K3* through ASK1) and might indeed represent downstream effectors of IGF‐PI3K‐AKT activation. The p27 protein encoded by the *CDKN1B* gene is a particularly attractive target since it is already known to represent a cancer driver due to its strong involvement in cell cycle arrest [22]. However, the functionality of p27 is tightly regulated at the post‐translational level and further characterisation was beyond the scope of this study.

Combining all results obtained by DNA sequencing, RNA sequencing and FISH, we conclude that *IGF1R* CN gain affected 18/253 osteosarcomas (7.1%). This genomic alteration resulted in significant overexpression of *IGF1R* and one of its main downstream signalling pathways (PI3K/AKT).

## Discussion

The prognosis for patients with osteosarcoma, particularly for those that are resistant to current treatment regimens and/or develop metastatic spread, is still dismal. As a consequence, new and innovative therapeutic approaches are urgently needed. Behjati *et al*. identified recurrent alterations of the IGF family of genes in a subgroup of osteosarcomas, comprising 7% of cases assessed by DNA sequencing (CN variation and truncating indels, *n* = 112) and up to 14% of cases evaluated by FISH (*n* = 87) [16]. Although the spectrum of mutations differs, we report a similar proportion of IGF alterations detected by DNA sequencing (6.7%, *n* = 134). The detection rate of *IGF1R* rearrangements by FISH, however, was lower in our set of tumours (9.2%, *n* = 119). The difference might be explained by the criteria used to define a gene amplification. In the study by Behjati and colleagues, more than 15 gene specific signals per cell were considered an *IGF1R* amplification, regardless of the number of centromeric control signals. In our study we used a ratio > 2 comparing gene and control probe signals.

One of the most consistent features of osteosarcomas lies in its numerous and complex structural rearrangements all over the genome. On average, 69 SCNVs with marked inter‐tumoral heterogeneity can be found per tumour [18]. The high amount of chromosomal instability increases the likelihood of abundant, randomly occurring and non‐functional passenger alterations, such as non‐functional *NTRK* gene fusions that we reported on only recently [23]. Discrimination of driver events from passenger mutations therefore is a critical step towards the identification of potential treatment targets and we believe that using a ratio between gene and control probes in *IGF1R* FISH analysis could be more reliable in identifying functionally relevant alterations. Indeed, our findings indicate that the *IGF1R* CN gains detected increased both its transcription and activated downstream signalling pathways. Although the identification of downstream effectors remains challenging in regards to the broad action spectrum of the PI3K/AKT signalling pathway, the p27 protein encoded by the *CDKN1B* gene appears to represent the most promising target. In contrast to *MYC*, *CDKN1B* is differently expressed and recurrently amplified among the tumours also showing an amplification of *IGF1R*. The p27 protein has contradictory roles in both promoting and inhibiting cell cycle progression, strongly dependent on its phosphorylation induced by AKT [22]. Additional studies are required to better characterise the phosphorylation status and functional impact of p27 in patients with osteosarcoma.

As an important trigger of tumour growth through its interaction with the PI3K‐AKT pathway, a therapeutic strategy against IGF1 and its receptor has raised hope for individual treatment approaches. Unfortunately, IGF1R inhibitors have largely proven inefficient in more than 70 clinical trials on various tumour subtypes [12]. Although the drugs efficiently interfere with the IGF1R transduction cascade, adaptive responses involving the insulin receptor or the EGFR signalling pathway have been reported to underlie the clinical treatment failure [11]. Indeed, the IGF1 receptor has long been considered the only active mediator of IGF1 and IGF2 signalling. Even after growing evidence indicated the presence of alternative insulin receptors to mediate IGF1 and IGF2 effects in the late 1990s, strategies to inhibit the IGF axis remained focused on IGF1R. Later, it was demonstrated that both IGF ligands could initiate a mitogenic response *via* hybrid receptors such as IGF1R/IR or IGF1R/EGFR. Even in the absence of IGF1R, activation of the RAS‐MAPK–ERK signalling cascades can still be triggered by IGF2 through a specific isoform of the insulin receptor (IR^A^) [14, 24]. Therefore, any therapeutic strategy solely targeting IGF1R or IGF1R/EGFR and/or IGF1R/IR (main isoform) is likely insufficient to prevent the activation of downstream signalling pathways.

A clinical trial (NCT00617890) evaluating the efficacy of an IGF1R antibody (Robatumumab) on bone sarcomas, including osteosarcomas, has already been carried out without any evidence of clinical benefit [10]. However, the participants were not stratified according to underlying alterations of the IGF signalling pathway which makes it impossible to rule out a potential treatment effect in patients with IGF‐mutated tumours. When activation of the IGF axis is a consequence of upstream stimulation, targeting IGF1R will most likely not suffice to effectively block downstream signalling cascades as the overexpressed ligands will be redirected to alternative receptors. This should not be the cases if the *IGF1R* gene alteration represents the oncogenic driver itself as suggested in the osteosarcomas presented here. Although escape mechanisms could still develop in these patients, improved drug responses were observed when the tumour growth specifically relied on the activation of IGF signalling pathways as shown in patients with Ewing sarcoma [15]. It would be intriguing to re‐evaluate tumour samples from patients who participated in previous clinical trials and correlate the response to treatment in the context of IGF pathway alterations.

Taken together, we believe that any targeted treatment approach in a highly rearranged tumour like osteosarcoma requires individual molecular work‐up with particular consideration of the potential functionality of individual treatment targets. In selected patients, IGF blockers might become a promising treatment supplement independent of the rather discouraging results reported so far.

## Author contributions statement

BA had full access to all the data in the study and takes responsibility for the integrity of the data and the accuracy of the data analysis. DB, BA, MK and MB conceived and designed the study. BA and MK acquired, analysed or interpreted data. BA and DB drafted the manuscript. AHK and DB revised the manuscript for important intellectual content. MN, AHK, OW and DB provided administrative, technical or material support. DB, MK and MN supervised the study.

## Supporting information


**Figure S1.** Oncoplot of the PI3K‐AKT–MTOR pathway
**Figure S2.** Oncoplot of the MYC Targets v2 pathway
**Figure S3.** Analysis of recurrent copy number variations using GISTIC2.0
**Figure S4.** Differential gene expression according to IGF1R copy number gains

**Table S1.** Clinical metadata and sequencing analysis metrics
