# Supplementary material for: Overactivation of the IGF signalling pathway in osteosarcoma: a potential therapeutic target?
Source: J Pathol Clin Res. 2020 Dec 8;7(2):165–72. doi: 10.1002/cjp2.191 (PMC7869926; doi:10.1002/cjp2.191)
Supplement: Supplementary file 1 — Figure S1. Oncoplot of the PI3K‐AKT–MTOR pathway Figure S2. Oncoplot of the MYC Targets v2 pathway Figure S3. Analysis of recurrent copy number variations using GISTIC2.0 Figure S4. Differential gene expression according to IGF1R copy number gains [file CJP2-7-165-s001.docx]

**Overactivation of the IGF signalling pathway in osteosarcoma: a potential therapeutic target?**

B Ameline *et al. J Pathol Clin Res* DOI: 10.1002/cjp2.191

**Supplementary Figures S1 – S4**

**
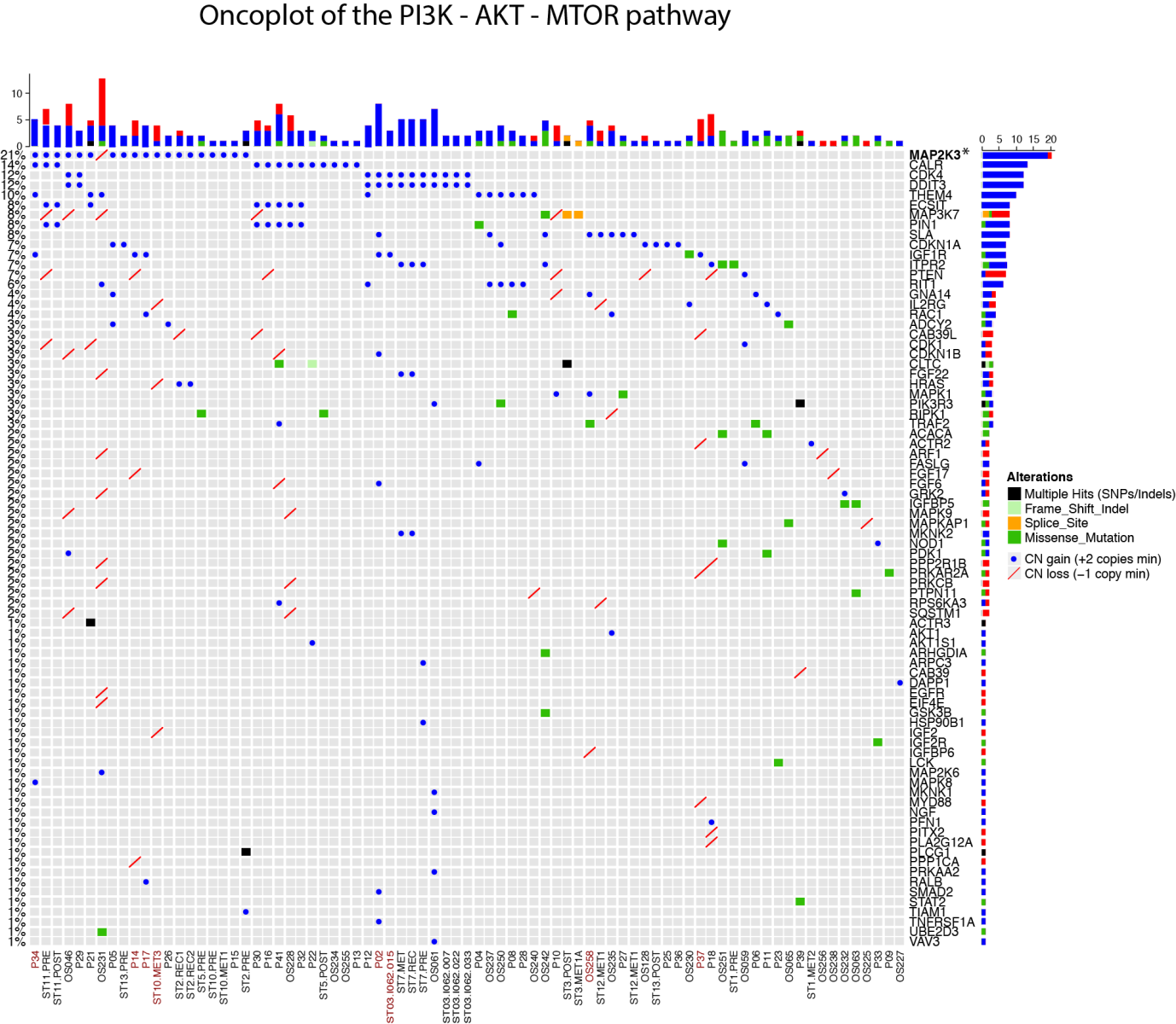
**

**Figure S1. Oncoplot of the PI3K-AKT-MTOR pathway.**

All 105 genes belonging to the gene set "Hallmark_PI3K_AKT_MTOR_Signaling" and the IGF family of genes (*IGF1, IGF1R, IGF2, IGF2R, IGFBP1, IGFBP2, IGFBP3, IGFBP4, IGFBP5, IGFBP6*) were investigated for SCNVs and SNVs in a cohort of 96 osteosarcomas (WES). IGF-altered samples have their names in red. Samples and genes without a single alteration were removed from the graph. **MAP3K2* alterations are significantly more frequent (Fischer’s exact test, p-value = 0.03) among the patients presenting an alteration of the IGF family of genes.


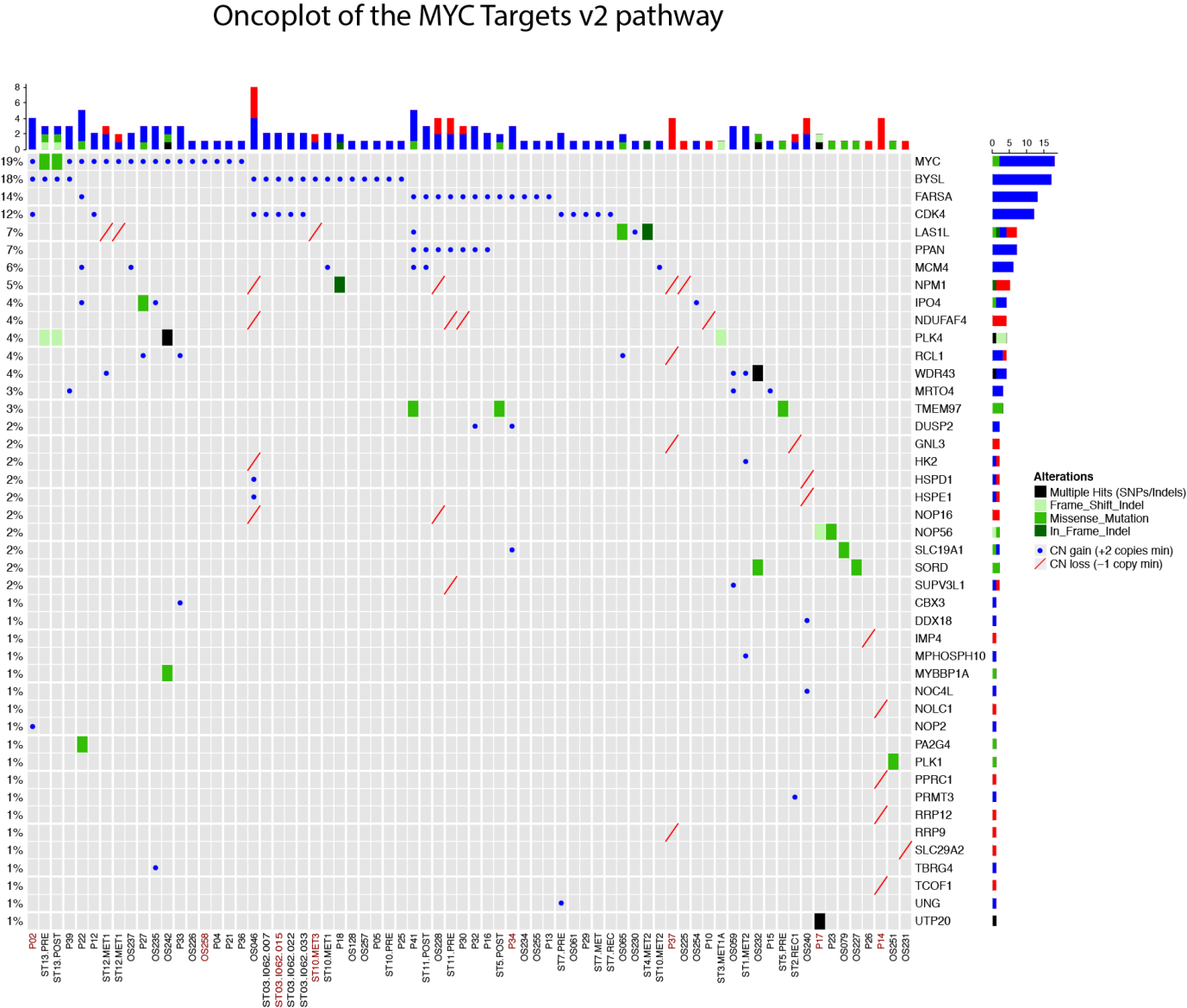


**Figure S2. Oncoplot of the MYC Targets v2 pathway.**

All 58 genes belonging to the gene set "Hallmark_MYC_TARGETS_v2" were investigated for SCNVs and SNVs in a group of 96 osteosarcomas (WES). IGF-altered samples have their names in red. Samples and genes without a single alteration were removed from the graph.

**
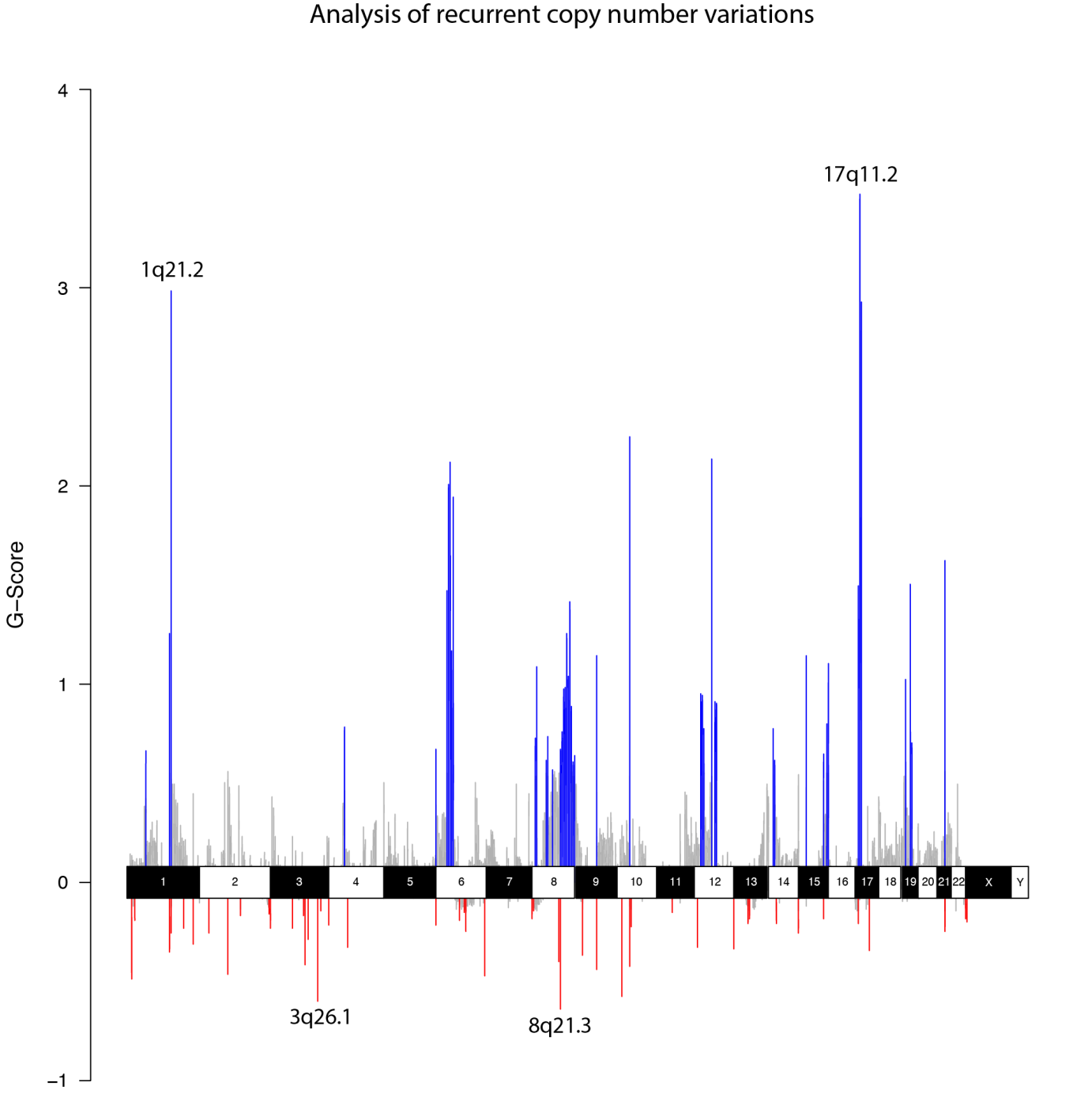
**

**Figure S3. Analysis of recurrent copy number variations using GISTIC2.0.**

Identification of recurrent somatic copy number gains (blue) and losses (red) in a cohort of 134 osteosarcomas performed with the GISTIC2.0 module. The G-score is deﬁned as the amplitude of the copy number multiplied by its frequency across samples. All event with a false rate recovery superior to 0.05 are coloured in grey.

**
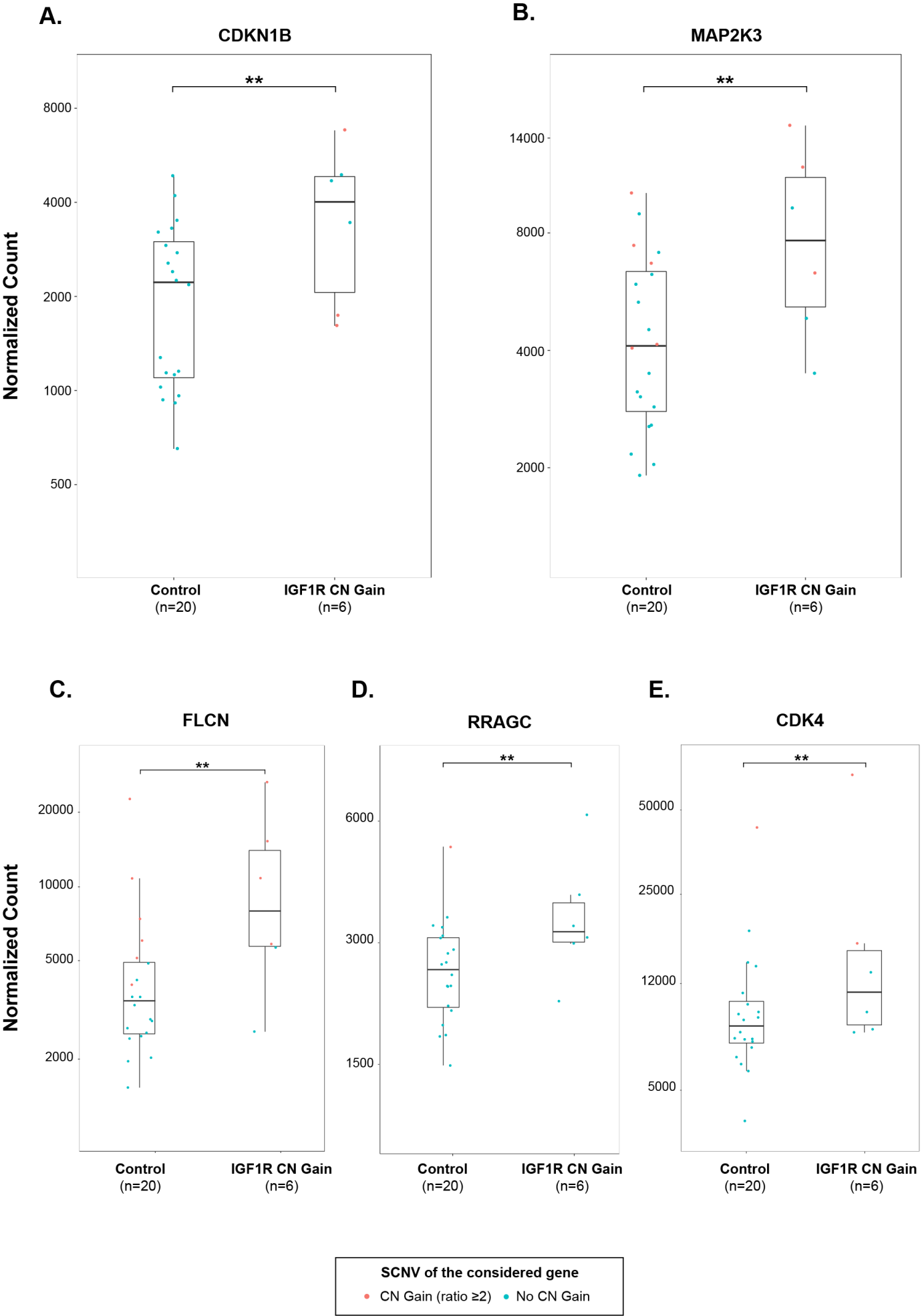
**

**Figure S4. Differential gene expression according to *IGF1R* copy number gains.**

Comparing the transcription of (A) *CDKN1B* gene, (B) *MAP2K3* gene, (C) *FLCN* gene, (D) *RRAGC* gene or (E) *CDK4* gene in tumours with (n=6) and without (n=20) *IGF1R* amplification revealed a statistically significant association using a Wald's test (p-value < 0.05; FDR > 0.05; Details in supplementary material, Table S1). Samples presenting a copy number gain (ratio ≥ 2) of the considered gene are coloured in red instead of green.
